# Psychometric properties of the self-efficacy scale for chronic disease management (SEMCD-S) in older Colombian adults

**DOI:** 10.1186/s40359-023-01347-4

**Published:** 2023-09-30

**Authors:** Lorena Cudris-Torres, Stefano Vinaccia Alpi, Álvaro Barrios-Núñez, Natali Gaviria Arrieta, Martha Luz Gómez Campuzano, Giselle Olivella-López, Juan Hernández-Lalinde, Valmore Bermúdez, Olaiza Lobato Pérez, Jorge Armando Niño-Vega, Jorge Navarro-Obeid, Román José Ortega Fernández, José Julián Javela

**Affiliations:** 1 Universidad de la Costa, Barranquilla, Colombia; 2 Universidad del Sinú, Montería, Colombia; 3 Clínica General del Norte, Barranquilla, Colombia; 4 Universidad Popular del Cesar, Valledupar, Colombia; 5 Fundación Universitaria del Área Andina, Valledupar, Colombia; 6 Universidad Simón Bolívar, Barranquilla, Colombia; 7 Centro de Investigaciones en Ciencias de la Vida, Universidad Simón Bolívar, Barranquilla, Colombia; 8 Universidad Pedagógica y Tecnológica de Colombia, Duitama, Colombia; 9 Corporación Universitaria del Caribe (CECAR), Sincelejo, Colombia

**Keywords:** Self-efficacy, Chronic diseases, Psychometric properties, Older adults, Colombia

## Abstract

**Background:**

Self-efficacy alludes to personal competence in an individual’s effectiveness when facing stressful situations. This construct has been related to different domains of the health field, finding that high levels of self-efficacy benefit human functioning and enhance well-being.

**Methods:**

The present study aimed to determine the psychometric properties of the self-efficacy scale for managing chronic diseases (SEMCD-S) by assessing factorial, convergent and divergent validity, reliability, and measurement invariance. Likewise, the comparison of self-efficacy according to socio-demographic characteristics was proposed by contrasting latent factors. An instrumental, transactional, descriptive, and non-experimental design study was carried out with the participation of 325 Colombian senior citizens.

**Results:**

The findings suggest that the scale has appropriate psychometric properties. The one-factor structure exhibited a satisfactory fit, the mean-variance extracted reported acceptable figures and the correlation analysis with other constructs supported this instrument’s convergent and discriminant validity. Likewise, it was invariant to the different socio-demographic aspects examined, while the internal consistency figures were high. Differences in the means of the latent factors were only detected in the academic grade. In this case, older adults with a primary school level attained higher self-efficacy values than those who had completed high school or university studies.

**Conclusions:**

It is concluded that the self-efficacy scale for chronic disease management is a valid and reliable instrument that can be used in the Colombian context to measure and compare this construct.

**Supplementary Information:**

The online version contains supplementary material available at 10.1186/s40359-023-01347-4.

## Background

General self-efficacy is understood as a cognitive construct that refers to the evaluation of the ability to perform actions with optimal performance [1]; that is, it is the beliefs that the individual has about his or her abilities to perform the tasks that the context demands [2]. Self-efficacy is considered an important concept in health psychology and is identified in this field as a predictor of health-promoting behaviours, being a factor that reduces harmful behaviours and effectively implements health promotion and disease prevention programmes, encouraging treatment adherence, and helpful in public health campaigns [3].

Self-efficacy has been studied in association with various behaviours in different health domains, forward chronic diseases, diet, physical activity, sexual behaviour, psychoactive substance use, weight reduction and ability to overcome health difficulties or to prevent health risk factors [4, 5]. Research agrees that high levels of self-efficacy promote people’s general well-being, enhancing overall health. For this reason, recognising and assessing self-efficacy in individuals is an essential factor in behaviour change, and interventions based on the self-efficacy model show greater effectiveness in promoting adherent behaviours [6, 7].

Given the benefits of self-efficacy, it is necessary to have instruments to measure and identify it. Psychometric or instrumental studies have evaluated short and robust scales whose findings show adequate functioning in countries in North America, Europe, Asia and South America, which so far do not include Colombia, the country has a high percentage of people with chronic diseases and low schooling, in addition to the fact that public policies designed for primary health care do not include psychoeducation that can contribute to patients having a better understanding of their disease and the care it requires to have a better quality of life [8].

Concerning how to measure self-efficacy in chronically ill patients, which is the specific focus of this research, in 2014, the Self-Efficacy Scale for the Management of Chronic Diseases SEMCD was designed, which was psychometrically validated with 6 items in English and 4 items in Spanish that measures respondents’ confidence in their ability to control fatigue, pain, emotional discomfort and other symptoms associated with the impact of the disease, and to carry out tasks and activities. It was designed using a 10-point numerical Likert scale ranging from 1, representing no confidence at all, to 10, indicating complete confidence. It is noted that higher scores reflect higher self-efficacy [9].

The Spanish version of the SEMCD scale was piloted with a sample of 67 Spanish speakers suffering from chronic illnesses. The results showed that two of the six items, specifically the fifth and sixth, were less favourable than the others on the overall scale. Thus, Cronbach’s alpha decreased slightly from 0.93 for four items to 0.89 when all six items were entered, and the two items showed lower to higher scale correlations of 0.46 and 0.67, respectively, compared to 0.81 to 0.86 for the other four items.

Thus, a four-item version was used to evaluate the Spanish-speaking chronic disease self-management programme (Taking Control of Your Health) and has subsequently been used in other studies [9]. All translations are valid and reliable [9]. The scales demonstrated high internal consistency reliability (Cronbach’s alpha) in all independent studies. Internal consistency for each sample ranged from 0.88 to 0.91 for the SEMCD. The mean of the four-item SEMCD-S was slightly higher at 6.2 for the two Spanish studies. Reliability for the SEMCD-S was 0.95 and 0.94 in the two samples.

The 6-item SEMCD scale has been psychometrically evaluated in non-US contexts such as Brazil [10], Germany [11], Iran [12], South Korea [13], Portugal [14], Turkey [15], Sweden [16], France [17], Saudi Arabia [18] and Canada [19].

García et al. [20] validated the 6-item SEMCD Scale in Spain in 135 patients with metabolic syndrome, whose mean age ranged from 55.5 years. The results indicate acceptable levels of validity and reliability in patients with MS. It helps measure the relationship of self-efficacy related to physical exercise with different psychosocial and lifestyle variables. The internal consistency of the scale was 0.925 and 0.864, according to Cronbach’s alpha and Guttman’s two halves method.

For their part, Nazar Carter et al. [21] carried out a study in Chile in which they evaluated the adherence to treatment in 141 patients diagnosed with hypertension in a health centre; among the instruments used, they included the 6-item SEMCD, which they obtained a Cronbach’s alpha of α = 0.791.

Studies developed by Lorig et al. [22], using the self-efficacy scale, found a Cronbach’s alpha ranging from 0.77 to 0.91, indicating acceptable internal consistency [23]. Sometime after the design of the original scale, other Spanish-language self-efficacy scales were developed by translating questions into Spanish and then confirming them with earlier translations. Conversely, what happened with English questionnaires motivated the reduction of the items on the scale, decreasing the burden of the questionnaire on the respondent.

Although the 4-item SEMCD scale has been validated in Spanish with the Latino population residing in the United States, the literature search did not find any studies on its validation in Latin American countries and other Spanish-speaking countries such as Spain, the Philippines and Equatorial Guinea, so its validation in the Colombian population will add current scientific knowledge and will serve as input for new psychometric studies.

Another variable studied in this research is the sociodemographic data that allow complementing the analysis of the population, according to several factors, among them: age, gender, marital status, schooling, with whom they live and the disease suffered by the patients. These studies have been carried out in different parts of the world by validating the SEMCD coping scale, mostly by validating 6 items and complementing their analyses with variables of age, gender, schooling, type of disease, among other cultural factors.

In Germany it was evidenced that the unidimensional component of the instrument according to sociodemographic data where age and gender, are aligned with greater chronic disease, through a score of SES6G, standardized − 0.27, P < .001. contrary to the educational level that its score does not determine a significant effect [11].

The validation carried out in Korea [13] shows how sociodemographic data are evaluated through the association with quality of life and disease, identifying that for diseases such as arthritis and asthma the quality of life is lower than for patients with hypertension, an analysis that is achieved thanks to the validation of sociodemographic data that evaluate age, gender, disease and support in symptom management. Similar to the validation carried out in Sweden, where sociodemographic data are collected to analyze the quality of life in patients with chronic diseases, with which the scale is validated [16].

It is important to mention a study carried out in Chile where self-efficacy is contrasted with sociodemographic variables and it was found that men have greater adherence than women to follow indications, but women have greater control in the consumption of medications, while at the level of education there was a significant difference related to the level of study, the greater the knowledge, the better the management of the disease, the greater the adherence to treatment and it is reflected in the quality of life [24].

Therefore, the following research aimed to determine the psychometric properties of the self-efficacy scale for managing chronic diseases in Colombian older adults by assessing factorial, convergent and divergent validity, reliability, and measurement invariance.

## Methods

### Type of research and design

In order to respond to the first research objectives, an instrumental design was implemented. According to Ato et al. [25], the validation and calculation of the psychometric properties of measurement instruments fall within this typology. Additionally, for the achievement of the second research objective, transactional-descriptive research with a non-experimental design was used [26].

### Participants

The study included 325 elderly people with chronic diseases, aged between 60 and 78 years, with a mean and standard deviation of 68.39 and 4.15 years (CV = 6.07%), respectively, residing in the Colombian Caribbean Region, 133 in the city of Valledupar (40.9%), 119 in Barranquilla (36.6%) and 73 in Chimichagua Cesar (22.5%); 174 were male (53.5%) and 151 were female (46.5%), there were significant differences between males and females for this characteristic (t = 3.31, df = 323, p < .001). Specifically, females presented an age of 69.19 years, with a standard deviation of 4.36 (CV = 6.30%), while males had a mean of 67.69 years, with a deviation of 3.83 (CV = 5.66%). Regarding marital status, 145 participants were married (44.6%), 99 were in union (30.5%), 62 were single (19.1%), 15 were widowed (4.6%) and 4 were separated or divorced (1.2%); it should be noted that three quarters of the participants lived with their partner. In terms of socioeconomic level, 169 belong to stratum 1 (52%), 148 to stratum 2 (45.5%) and 8 to stratum 3 (2.5%), which implies that the absolute majority (97.5%) of the sample is in the lower socioeconomic strata, the most vulnerable. In terms of schooling level, 91 completed primary education (28%), 80 did not complete primary school (24.6%), 77 completed secondary education (23.7%), 61 did not complete secondary education (18.8%) and 16 participants did not complete university studies (4.9%); none of the participants had professional training. In half of the cases (52.6%) they were not able to go beyond basic primary education.

### Procedure

The research involved the participation of 325 chronically ill older adults. Individuals were selected by non-probability purposive sampling based on the following criteria: (1) persons of any sex, gender, sexual orientation or preference; (2) aged at least 60 years; (3) diagnosed with chronic disease of at least one year of antiquity verified with the institutional medical history.; (4) with no evidence of cognitive impairment identified through scores greater than 23 on the Mini-Mental State Examination (MMSE); (5) with normal literacy skills verified by direct questioning and subsequently by reading and signing the informed consent form.; and (6) without special conditions that could limit their participation in the study such as: physical disability, functional limitation, supplemental oxygen requirement, among others detectable at the time of the survey. No equations were used to calculate the sample size because of non-probability sampling. Therefore, the number of people was set by their willingness to participate, by logistical and budgetary constraints, and by the researchers’ intention to have the number of people meet the literature recommendations. Consequently, the 325 older adults constitute a sample that complies with the standard recommendation of 10 subjects per item [27] but with less conservative suggestions [28–31].

Potential participants were approached in the outpatient waiting room of the Clínica General del Norte, the general aspects of the study were explained to them and they were invited to participate in the study, the people who expressed their willingness to participate in the research signed the informed consent form, authorizing them to review the clinical history, as well as their personal data and home address to be surveyed.

The study was conducted in the first quarter of the year 2022, at which time the use of masks and hand washing in hospital environments was mandatory.

First, a pilot study was carried out to identify if there were any words, items or questions that were difficult for the participants to understand, as well as to assess the older adult’s ability to understand and to determine the response time for each scale. This phase was used to check that the administration of the instruments was easy and to plan the final stage of data collection. The older adults who formed part of the sample were surveyed in their homes, which were in the departments of Cesar and Atlántico in Colombia, considering the criteria mentioned above. Before applying the scales, the general aspects of the work were communicated, emphasising that participation was completely voluntary and that it did not involve risks to the mental, physical, or emotional health of those involved. It was also emphasised that the information collected would be used for research purposes only, stressing that participants could leave the study at any time without violating their integrity. Subsequently, informed consent was signed, after which a form was provided with the socio-demographic data used in the analysis. Then the MSSE was applied to detect cases of possible cognitive impairment. Finally, all the instruments necessary to meet the research objectives were administered. It should be noted that the procedure was developed following the recommendations of the American Psychological Association (APA) but also respecting the guidelines of the Colombian College of Psychologists (COLPSIC). The study was approved by the Ethics Committee of the Clínica General del Norte in Barranquilla, Colombia, approved in the session of 21 December 2021.

No personal data were recorded to identify the participants; the information in the instruments was handled by codes.

### Instruments

The Self-Efficacy Scale for the Management of Chronic Diseases (SEMCD-S) was the main instrument:

#### Self-Efficacy Scale for the management of chronic diseases (SEMCD-S)

This scale was initially developed by Loring [32] in research work undertaken to find out how people cope with chronic illness. As such, the SEMCD-S was developed to measure an individual’s confidence in their personal resources and ability to cope effectively. Most instrument versions have six items constructed in a Likert-type format but with 10 response options ranging from 1 (totally unsure) to 10 (totally confident). However, there are alternatives for four items that resulted from work carried out with Spanish speakers. Ritter and Lorig [9] describe the process of adapting the scale to Spanish, indicating that the elimination of the fifth and sixth items improved Cronbach’s alpha coefficient from 0.89 to 0.93 and increased the practicality of the instrument by having a less extensive option. In this research, the Spanish version of the SEMCD-S was administered, consisting of four statements.

#### Emotional regulation questionnaire (ERQ)

The ERQ was designed to measure two emotional regulation strategies: cognitive reappraisal and expressive suppression [33]. It is a scale of 10 seven-choice Likert-style statements ranging from 1 (strongly disagree) to 7 (strongly agree). Cognitive reappraisal comprises items 1, 3, 5, 7, 8 and 10, while suppression comprises items 2, 4, 6 and 9. Regarding psychometric properties, Gross and John [34] found reliability figures measured by Cronbach’s alpha coefficient ranging from 0.75 to 0.82 in independent samples. Furthermore, the authors validated the two-dimensional structure of the scale through confirmatory factor analysis by comparing four theoretically plausible models. They also established convergent and discriminant validity through correlation analysis with constructs such as success regulation, inauthenticity, coping and mood regulation, extended personality, impulse control, cognitive ability and desirability.

#### Fatigue severity scale (FSS)

The Fatigue Severity Scale (FSS) is one of the most widely used scales to measure fatigue in people with chronic diseases. It was developed by Krupp et al. [35] as a result of a study working with individuals with multiple sclerosis. It is designed as a nine-item Likert-type inventory with seven response alternatives ranging from 1 (strongly disagree) to 7 (strongly agree), grouped into three factors: physical impairment, social impairment and motivational impairment. According to several studies, the instrument’s internal consistency is appropriate, with Cronbach’s alpha coefficients ranging from 0.81 to 0.94 [36–38]. Evidence of temporal stability has also been found through test-retest analyses [41], convergent and discriminant validity by analysing the correlation with similar scales, and questionnaires measuring opposite constructs [39].

#### Medical outcomes study sf-36 health questionnaire (MOS SF-36)

The «Short Form Health Survey» (SF-36) is part of the Medical Outcomes Study. This observational research study has been conducted since 1989 and has evaluated more than 20,000 people with chronic diseases using transactional and longitudinal designs [40]. It is a scale that can be used to obtain a patient’s health profile in general populations and subgroups. This instrument is composed of 36 Likert-style questions with non-uniform response options that vary in number and definition, which are grouped into eight dimensions: physical function, physical role, bodily pain, general health, vitality, social function, emotional role and mental health. Multiple research reports good psychometric properties of the SF-36 questionnaire, which has been successfully translated into Spanish [41, 42].

The reliability of the MOSSF-36 Health Questionnaire is 0.7; the physical role, physical function and emotional role measures obtained reliability scores equal to 0.90; in terms of content validity they presented high correlations with the physical component (r ≥ .74).

### Statistical analysis

First, a database scan was carried out to identify anomalies that could be due to transcription errors, a procedure in which no faults were found. Subsequently, descriptive analysis of the items was carried out to ascertain the centralisation and dispersion of the data but also to verify aspects such as normality and the existence of atypical data, which was checked at both the one-variate and multivariate levels. For this, the Shapiro-Wilk and Mardia tests were used, as well as Q-Q plots, box plots and robust Mahalanobis distances. It was corroborated at this stage that the violation of the normality assumption was significant and that the presence of outliers was moderate.

The structure of the scale was evaluated using Confirmatory Factor Analysis (CFA). In this sense, the maximum likelihood method with robust standard errors and the adjusted statistic was chosen, an appropriate strategy to work in situations where there is an absence of normality, the presence of outliers and instruments with at least seven response options (Satorra & Bentler, 2001). The fit was examined using traditional tools: chi-square statistic (χ^2^), standardised chi-square statistic (χ^2^/*df*), comparative fit index (CFI), Tucker-Lewis fit index (TLI) and root mean standardised residual (SRMR). Root means the square error of approximation (RMSEA) was not used due to the simplicity of the models tested where there are few degrees of freedom. Simulation studies have found that RMSEA significantly underestimates the fit, even when the difference between the covariance matrices is minimal and the chi-square test is insignificant [43].

Factor adequacy was assessed through the significance of the chi-square test, but it was also taken into account that the standardised chi-square statistic was less than or equal to 3.00 due to the sample size. The cut-off point for the CFI and TLI was 0.95, while the benchmark for the SRMR was 0.05 [44, 45]. Convergent validity was established by the Mean-Variance Extracted (MEV), which, to corroborate this property, should be equal to or greater than 0.50 [46]. Correlation analysis was performed to complement the evidence of convergent and discriminant validity, using the Spearman-Brown Rho coefficient given the behaviour of the data [47, 48]. Reliability was inspected employing Cronbach’s alpha and McDonald’s omega coefficients, classifying these figures according to the following criteria: unacceptable (0.50), poor (0.60), questionable (0.70), acceptable (0.80), good (0.90) and excellent (0.95).

In the invariance test, the nested models resulting from imposing successive restrictions on factor loadings (metric) and intercepts (scalar) were compared, taking as a reference the model in which the overall structure had to be the same in each group (configurational). The scale was confirmed to be invariant if the difference in the chi-squared statistics was non-significant, using the methodology proposed by Satorra and Bentler [49]. As a complementary tool, the difference in the CFI of the contrasted models was calculated, assuming differences equal to or less than 0.01 as satisfactory [50]. Equivalence was tested for all socio-demographic aspects of the paper, except for the question of medication for the patient’s chronic disease, because all answered affirmatively. Therefore, the analysis of invariance considered sex, municipality of residence, marital status, companion, academic level or degree, socioeconomic stratum, health system and use of psychiatric medication.

The means of the latent factors according to socio-demographic variables were compared with structural equations using confirmation factor analyses in which both the structure of variance-covariance matrices and the structure of means were included. In contrast, the reference group’s mean and variance were set to zero and one, respectively, while the parameters corresponding to the other groups were freely estimated. It allowed for a Wald test using the Z-statistic. It should be noted that the selection of the reference group was arbitrary. Additionally, the effect size was estimated using Cohen’s d coefficient but adjusting the differences to the context of multigroup CFA according to Hancock’s recommendations [51]. Finally, data processing and analysis were performed with IBM SPSS and various R-Studio libraries such as Lavaan, SemTools, SemPlot, Psych, MVN and Mvoutliers. Significance was set at values less than 0.05.

## Results

### Descriptive analysis of the items

The analysis of the items is shown in Table 1. As can be seen, the average score ranged from 7.71 to 8.37, implying a grand mean of 8.11. In terms of dispersion, the values ranged from 1.19 to 1.34, with an average standard deviation of 1.27. The sum of the items reflected a mean of 32.42 points, with a standard deviation of 4.18, indicating that the participants’ self-efficacy was high. The standardised skewness and kurtosis were considerably high, allowing us to rule out the assumption of normality in a first descriptive approximation. At the inferential level, the Shapiro-Wilk statistics were significant, findings which, together with those obtained in the Mardia test and not shown in Table 2 (Mardia g_1_ = 1018.81, p < .001, Mardia g_2_ = 49.98, p < .001), lead to the complete rejection of this assumption. Furthermore, the reliability indices suggest that the scale has good psychometric properties concerning this characteristic.

**Table 1:** Descriptive analysis of the Spanish version of the SEMCD-S

Items and description	M	SD	*Z*_*g*1_	*Z*_*g*2_	SW	α	λ_6_	ITCC
**IT01S.** ¿Qué tan seguro se siente Ud. de poder evitar que la fatiga o el cansancio debido a su enfermedad interfiera con las cosas que quiere hacer?	8.36	1.24	-19.57	27.93	0.57^***^	0.81	0.76	0.66
**IT01E**. How confident do you feel that you can keep the fatigue caused by your disease from interfering with the things you want to do?								
**IT02S**. ¿Qué tan seguro se siente Ud. de poder evitar que las dolencias debido a su enfermedad interfieran con las cosas que quiere hacer?	7.71	1.34	-9.89	6.87	0.82^***^	0.81	0.74	0.67
**IT02E**. How confident do you feel that you can keep the physical discomfort or pain of your disease from interfering with the things you want to do?								
**IT03S**. ¿Qué tan seguro se siente Ud. de poder evitar que el estado emocional debido a su enfermedad interfiera con las cosas que quiere hacer?	7.98	1.29	-12.49	11.49	0.76^***^	0.77	0.70	0.74
**IT03E**. How confident do you feel that you can keep the emotional distress caused by your disease from interfering with the things you want to do?								
**IT04S**. ¿Qué tan seguro se siente Ud. de poder evitar que algunos otros síntomas o problemas de salud que tenga interfieran con las cosas que quiere hacer?	8.37	1.19	-19.41	28.25	0.58^***^	0.81	0.76	0.65
**IT04E**. How confident do you feel that you can keep any other symptoms or health problems you have from interfering with the things you want to do?								
**Total.** Items summation	32.42	4.18	NA	NA	NA	NA	NA	NA

M: mean. SD: standard deviation. *Z*_*g*1_: standardised skewness. *Z*_*g*2_: standardised kurtosis. SW: Shapiro-Wilk univariate statistic. α: Cronbach’s alpha unstandardised coefficient. λ_6_: Guttman Lambda 6’s unstandardised coefficient. ITCC: item-total corrected correlation. NA: not applied. ^***^p < .001

### Factorial validity

**Fig. 1:**
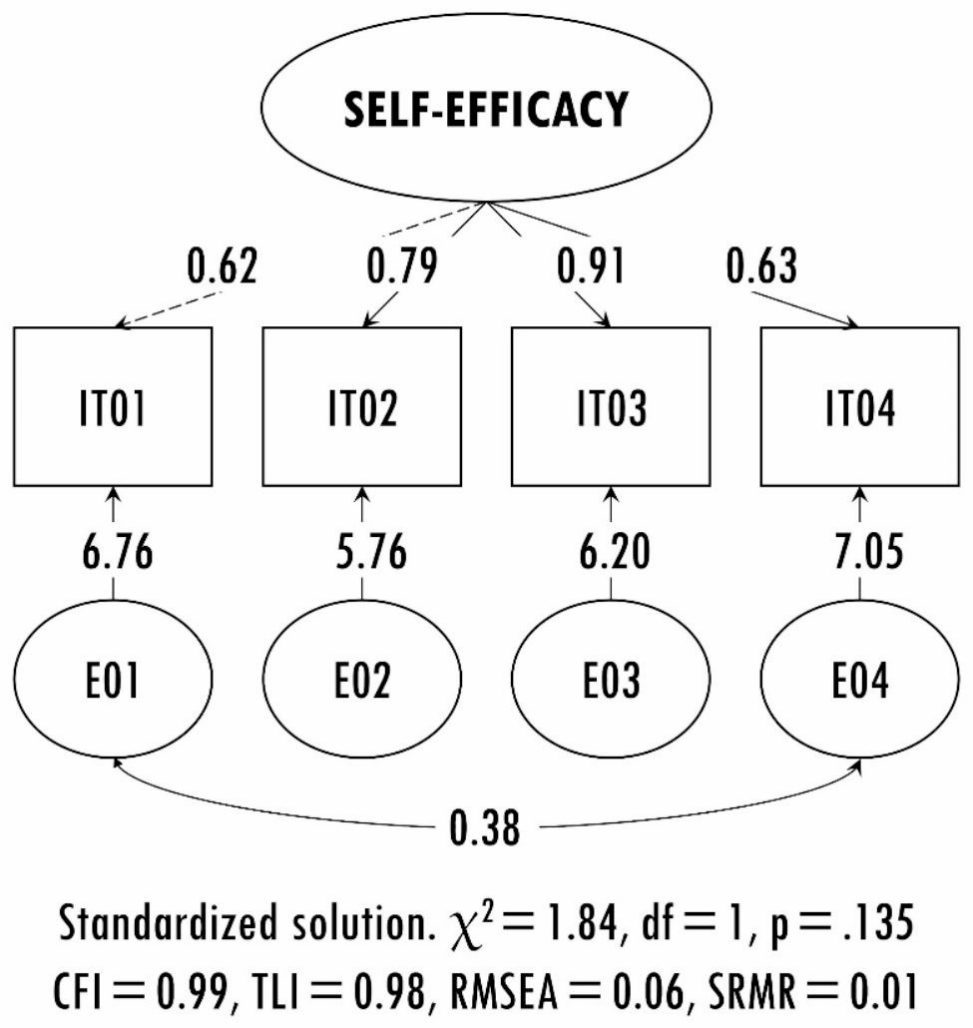
Standardised solution of the model proposed for the Spanish version of the SEMCD-S

This research did not replicate the unidimensional model of the self-efficacy scale in chronic patients. The chi-square test was significant (χ^2^ = 26.65, *df* = 2, *p* < .001) and the fit indicators exhibited values below the suggested cut-off points (χ^2^*/df* = 13.32, CFI = 0.89, TLI = 0.66, SRMR = 0.05). Because of this, the modification indices were explored, and it was found that the residual correlation of items 1 and 4 significantly improved the model fit. After evaluating this option, the chi-square test was found to be non-significant (χ^2^ = 1.84, *df* = 1, *p* = .175), and additional fit indicators reported optimal values (χ^2^/*df* = 1.84, CFI = 0.99, TLI = 0.98, SRMR = 0.01). It can be corroborated by Fig. 1.

### Convergent, divergent and reliability validity

The average variance extracted from the scale was 0.57, slightly higher than the recommended benchmark. The convergent and divergent validity is shown in Table 3. In this sense, it is worth noting that the correlations behave as expected, exhibiting positive values with the related variables of the study but negative values with those theoretically opposed constructs. Most of the findings in this phase report an association of moderate or high magnitude, which was presumed at the time of hypothesising the research. In terms of reliability, Cronbach’s alpha coefficient was 0.84 (95% CI: 0.78–0.89), while McDonald’s omega coefficient was 0.80 (95% CI: 0.76–0.86), implying satisfactory attributes.

**Table 2:** Convergent and divergent validity of the Spanish version of the SEMCD-S

Variable (scale)	Dimensions	Cronbach’s Alpha	Self-efficacy (SEMCD-S)		
			SB-Rho	95% LCL	95% UCL
**Emotional regulation (ERQ)**	Cognitive reappraisal	0,81	0.59^***^	0.51	0.66
	Expressive suppresion	0,74	0.27^***^	0.17	0.37
	Total	0,76	0.32^***^	0.21	0.41
**Fatigue severity (FSS)**	Emotional affectation	0,926	–0.66^***^	–0.72	–0.59
	Physic affectation	0,747	–0.56^***^	–0.63	–0.47
	Social affectation	0,846	–0.54^***^	–0.62	–0.46
	Total	0,87	–0.60^***^	–0.66	–0.52
**Quality of life (MOS SF-36)**	Physical functioning	0,89	0.30^***^	0.20	0.40
	Role limitations due to physical health	0,92	0.52^***^	0.43	0.60
	Pain	0,84	–0.27^***^	–0.37	–0.17
	General health	0,83	0.27^***^	0.16	0.37
	Energy/fatigue	0,79	0.55^***^	0.46	0.62
	Social functioning	0,89	0.17^**^	0.06	0.28
	Role limitations due to emotional problems	0,91	0.68^***^	0.61	0.73
	Emotional well-being	0,87	0.76^***^	0.71	0.80

SB Rho: Spearman-Brown’s Rho correlation coefficient. 95% LCL: Fieller-Harley-Pearson’s 95% lower confidence limit. 95% UCL: Fieller-Harley-Pearson’s 95% upper confidence limit. ^**^p < .01. ^***^p < .001

### Measurement invariance

The invariance according to various socio-demographic aspects such as gender, companion with whom the participant lives, economic stratum, residence, marital status and academic level is presented in Table 3. It can be verified that the difference between the chi-squared statistics of nested models compared was not significant, with the only exception of signs corresponding to the constants of intercepts according to the city of residence (Δχ^2^ = 12.88. *p* > .05). Although, the complementary tool based on the difference in CFI was very appropriate to the suggested value (ΔCFI = 0.011).

**Table 3:** Measurement invariance of the Spanish version of the SEMCD-S according to socio-demographic characteristics

Variable	Invariance	χ^2^ (*df*)	χ^2^/*df*	CFI	TLI	SRMR	Δχ^2^ (Δ*df*)	ΔCFI
**Sex**	Configural	4.873^†^ (2)	2.437	0.994	0.963	0.009	NA	NA
	Metric	5.876^†^ (5)	1.175	0.997	0.993	0.046	2.096^†^ (3)	0.003
	Scalar	8.177^†^ (8)	1.022	1.000	1.001	0.049	1.734^†^ (3)	0.003
**Town**	Configural	6.382^†^ (3)	2.127	0.993	0.959	0.014	NA	NA
	Metric	12.633^†^ (9)	1.404	0.989	0.978	0.078	7.217^†^ (3)	0.004
	Scalar	23.957^†^ (15)	1.597	0.978	0.973	0.088	12.884^*^ (3)	0.011
**Civil status**	Configural	6.357^†^ (3)	2.119	0.996	0.973	0.009	NA	NA
	Metric	11.327^†^ (9)	1.259	0.996	0.991	0.031	5.945^†^ (6)	0.000
	Scalar	15.515^†^ (15)	1.034	0.999	0.999	0.033	3.800^†^ (6)	0.003
**Who live with**	Configural	6.560^*^ (2)	3.280	0.993	0.957	0.008	NA	NA
	Metric	4.313^†^ (5)	0.863	1.000	1.004	0.011	0.150^†^ (3)	0.007
	Scalar	5.764^†^ (8)	0.721	1.000	1.007	0.013	1.100^†^ (3)	0.000
**Academic level**	Configural	3.725^†^ (2)	1.863	0.996	0.975	0.006	NA	NA
	Metric	4.827^†^ (5)	0.965	1.000	1.001	0.031	1.842^†^ (3)	0.004
	Scalar	5.796^†^ (8)	0.725	1.000	1.009	0.032	0.230^†^ (3)	0.000
**Economic level**	Configural	2.881^†^ (2)	1.441	0.998	0.986	0.008	NA	NA
	Metric	2.801^†^ (5)	0.560	1.000	1.020	0.027	0.611^†^ (3)	0.002
	Scalar	6.360^†^ (8)	0.795	1.000	1.008	0.033	4.804^†^ (3)	0.000
**Health system**	Configural	1.643^†^ (2)	0.822	1.000	1.006	0.008	NA	NA
	Metric	5.827^†^ (5)	1.165	0.998	0.995	0.039	4.112^†^ (3)	0.002
	Scalar	10.131^†^ (8)	1.266	0.995	0.992	0.038	4.530^†^ (3)	0.003
**Medication**	Configural	8.998^*^ (2)	4.499	0.991	0.945	0.008	NA	NA
	Metric	10.533^†^ (5)	2.107	0.991	0.980	0.015	2.621^†^ (3)	0.000
	Scalar	12.048^†^ (8)	1.506	0.994	0.990	0.015	1.688^†^ (3)	0.003

χ^2^: chi-square statistics. *df*: degrees of freedom. χ^2^/*df*: normed chi-square statistics. CFI: comparative fit index. TLI: Tucker-Lewis index. SRMR: standardised root means square residual. Δ: delta symbol indicating the difference between compared indices. Three decimal places have been used to increase accuracy. ^†^*p* > .05, ^*^p < .05

### Self-efficacy according to socio-demographic aspects

There were no differences in the self-efficacy for chronic disease management of the participants analysed in this study, except when this construct was compared concerning academic level. Note in Table 4 that, unlike this characteristic, the means of the latent factors were similar in the rest of the socio-demographic aspects. In this case, the results suggest that people with a bachelor’s degree or university studies exhibited higher self-efficacy than those who reached primary school levels, which implies a statistically significant difference of low magnitude (Dif.=0.38, *z* = 2.33, *p* = .020, Cohen’s *d* = 0.32).

**Table 4:** Differences in latent factors mean according to socio-demographic characteristics

Characteristic	Categories	*n (h)*	M (Var)	Cohen’s *d*
**Sex**	Male^a^	174 (53.54)	0.00_a_ (1.00)	NA
	Female	151 (46.46)	0.06_a_ (0.89)	
**Town of residence**	Valledupar^a^	133 (40.92)	0.00_a_ (1.00)	NA
	Chimichagua	73 (22.46)	–0.15_a_ (1.09)	
	Valledupar^a^	133 (40.92)	0.00_a_ (1.00)	NA
	Barranquilla	119 (36.62)	–0.08_a_ (1.15)	
	Chimichagua^a^	73 (22.46)	0.00_a_ (1.00)	NA
	Barranquilla	119 (36.62)	0.06_a_ (1.06)	
**Civil status**	Married or in free union^a^	244 (20.31)	0.00_a_ (1.00)	NA
	Single, separated or divorced	66 (75.08)	0.01_a_ (0.84)	
	Married or in free union^a^	244 (20.31)	0.00_a_ (1.00)	NA
	Widowed	15 (4.61)	–0.15_a_ (2.35)	
	Single, separated or divorced^a^	66 (75.08)	0.00_a_ (1.00)	NA
	Widowed	15 (4.61)	–0.18_a_ (2.81)	
**Who live with**	With family or friends^a^	279 (85.85)	0.00_a_ (1.00)	NA
	Alone	46 (14.15)	–0.18_a_ (1.29)	
**Academic level**	Elementary school^a^	171 (52.62)	0.00_a_ (1.00)	0.32
	High school or undergraduate	154 (47.38)	0.38_b_ (1.86)	
**Economic level**	Stratum 1^a^	169 (52.00)	0.00_a_ (1.00)	NA
	Stratum 2 or 3	156 (48.00)	–0.17_a_ (1.65)	
**Health system**	Subsidiary^a^	300 (92.31)	0.00_a_ (1.00)	NA
	Contributory	25 (7.69)	–0.13_a_ (1.30)	
**Psychiatric medication**	No^a^	313 (96.31)	0.00_a_ (1.00)	NA
	Yes	12 (3.69)	–0.15_a_ (0.78)	

*n*: count. *h*: percentage. M: latent factor means. Var: latent factor variances. *Z*: test statistics. *p*: *p*-value. Cohen’s *d*: Cohen’s *d* effect size measure

## Discussion

Self-efficacy has become a construct of great interest to researchers, as it involves essential elements in setting and achieving goals while allowing the identification of circumstances that influence the individual’s perception of his or her capabilities [52, 53]. In this sense, Bandura [1] considers that human beings have a system that allows them to exercise control over their inner self and the elements involved, such as feelings, thoughts, motivations and behaviours. It is this system that provides the individual with a series of reference mechanisms on which to regulate the process of perception and evaluation of one’s behaviour in such a way that how the results obtained from daily actions are interpreted functions as an input and can modify performance at a later level [54]. It is interesting, as Yang et al. [55] found, low self-efficacy is associated with an increased risk of worsening disease in chronic patients because chronic patients tend to increase pain intensity [56].

In coherence with this, the implications of effect and emotions in patients with chronic diseases have gained significant space in the scientific community, constituting a priority and a necessity for researchers who, through rigorous work, seek to establish and validate tools and measurement parameters that allow them to review the central topic of this study: self-efficacy [57, 58]. Therefore, the primary purpose of the present research was to determine the psychometric properties of the self-efficacy scale for chronic disease management by assessing factorial, convergent and divergent validity. The results suggest that, for descriptive validity, the reliability indices show that the scale has good psychometric properties. Similarly, for factor analysis, the chi-square test was significant with lower fit indicators than initially suggested, where there was a significant improvement in model fit for items 1 and 4, and an additional fit of optimal values was evident. Regarding the analysis according to socio-demographic aspects, there were no differences in the participants’ self-efficacy for chronic disease management in this study. However, subjects with high school or undergraduate studies showed higher self-efficacy than those with only primary school levels, factors that future researchers are recommended to review in multivariate studies.

The findings obtained indicate that the self-efficacy scale has adequate internal consistency in the sample participating in the study, which means that it has the psychometric properties to measure homogeneously an underlying construct of the items used. Consistent with this, internal consistency can be considered a congruent indicator to arrive at the conceptualisation of self-efficacy as a general construct [59–60].

Findings are congruent with those found by Riehm et al. [61], who assessed the validity and internal consistency reliability of the Self-Efficacy for Chronic Disease Management (SEMCD) scale in patients with systemic sclerosis, where they found that internal consistency was high and associations with measures of psychological and physical functioning were moderate to severe, confirming the initial hypothesis of their research. The researchers concluded that the scale scores are valid for measuring self-efficacy in patients with systemic sclerosis, confirming the use of the instrument to assess the effectiveness of self-care programmes.

Finally, although the literature review indicates that, to date, some scales have been designed for this purpose [4, 5]; however, the need arises to carry out a validation process that circumscribes this scale as a brief instrument for the evaluation of self-efficacy in patients with chronic diseases. In this way, we seek to leave an encouraging result because it represents a support instrument for the measurement and evaluation of self-efficacy that provides inputs for the design and implementation of intervention programmes for the management of chronic diseases in both patients and family members and caregivers.

There were no differences in the self-efficacy for the management of chronic disease of the participants analyzed in this study, except when this construct was compared in relation to academic level, in which 52% of the sample completed elementary school and 24% completed secondary school, see Table 4, which coincides with the research conducted in Chile where a relationship was found between educational level and adherence to treatment in patients with chronic diseases and stress [32], while educational schooling is not related to self-efficacy [19, 21, 24].

In contrast to this characteristic, in the rest of the sociodemographic aspects the means of the latent factors were similar, different from studies conducted in Europe and Asia where age and gender marked a significant difference in self-efficacy against the disease and quality of life of patients. [19, 24]; in this case, the results suggest that people who completed high school or university studies showed higher self-efficacy than those who reached primary school levels, which implies a statistically significant difference of low magnitude (Dif.=0.38, z = 2.33, p = .020, Cohen’s d = 0.32).

The 4-item SEMCD scale was used for the evaluation of the Spanish-speaking chronic disease self-management program and other studies [9]. All translations have been found to be valid and reliable [9]. The scales indicated high internal consistency reliability in all the different studies. The internal consistency for each sample ranged from 0.88 to 0.91 for the SEMCD. The mean of the four-item SEMCD-S was slightly higher at 6.2 for the two Spanish studies. The reliability for the SEMCD-S was 0.95 and 0.94 in the two samples. In the validation performed in the Colombian population with respect to reliability, the Cronbach’s alpha coefficient was found to be 0.84 (95% CI: 0.78–0.89), while the McDonald omega coefficient was 0.80 (95% CI: 0.76–0.86), implying satisfactory attributes.

### Limitations

As a limitation, it was not possible to establish the representativeness of the sample, because in Colombia there is no reliable and consolidated information on the exact number of people suffering from chronic diseases.

Considering that external financing was not available, participants from one of the five geographic regions of Colombia were included, which required more time to obtain the information.

## Conclusion

The self-efficacy scale provides critical elements for research and intervention processes in the area of health psychology and has appropriate psychometric properties for further study of this variable in subsequent studies since it reviews cognitive, emotional and social aspects, and therefore constitutes a basis for the study of self-efficacy in the management of chronic diseases.

The present study results show that the self-efficacy scale has an adequate internal consistency in the sample participating in this research, being a valid and reliable instrument among the Spanish-speaking population since the evaluation of consistency tested by Cronbach’s alpha showed that it obtained an excellent reliability index. In this sense, it is recommended that future studies, within the socio-demographic analysis, analyse cultural and socialisation differences that allow for establishing differences between the forms of self-efficacy perceived by the participants. Likewise, a multivariate analysis or a predictor model should be included to establish which factors are associated with self-efficacy in managing chronic diseases.

The study serves as input for research and intervention projects aimed at improving the health conditions of older adults with diseases. The validation of the SEMCD-S scale arose within the framework of the project “self-efficacy, perception of disease, emotional regulation and fatigue on the health-related quality of life of older adults residing in the departments of Cesar and Atlántico with a diagnosis of chronic disease”.

Finally, the findings also have important clinical and health assessment implications. It contributes to overcoming health difficulties and preventing illness, so it can be considered an appropriate clinical instrument to carry out the variable assessment. Self-efficacy is an essential indicator in the process of health and illness, as it enhances people’s overall health and well-being.

### Electronic supplementary material

Below is the link to the electronic supplementary material.

Supplementary Material 1

## Data Availability

The datasets used and analysed during the current study will be available from the corresponding author on reasonable request. The datasets used and/or analyzed during the current study are available from the corresponding author on reasonable request.
