# Supplementary material for: Psychometric properties of the self-efficacy scale for chronic disease management (SEMCD-S) in older Colombian adults
Source: BMC Psychol. 2023 Sep 30;11:301. doi: 10.1186/s40359-023-01347-4 (PMC10543854; doi:10.1186/s40359-023-01347-4)
Supplement: Supplementary file 1 — Supplementary Material 1 [file 40359_2023_1347_MOESM1_ESM.doc]

**Appendix A.** SEMCD-S Self-efficacy in chronic patients by Ritter and Lorig.

**IT01S.** ¿Qué tan seguro se siente Ud. de poder evitar que la fatiga o el cansancio debido a su enfermedad interfiera con las cosas que quiere hacer?

**IT01E.** How confident do you feel that you can keep the fatigue caused by your disease from interfering with the things you want to do?

**IT02S.** ¿Qué tan seguro se siente Ud. de poder evitar que las dolencias debido a su enfermedad interfieran con las cosas que quiere hacer?

**IT02E.** How confident do you feel that you can keep the physical discomfort or pain of your disease from interfering with the things you want to do?

**IT03S.** ¿Qué tan seguro se siente Ud. de poder evitar que el estado emocional debido a su enfermedad interfiera con las cosas que quiere hacer?

**IT03E.** How confident do you feel that you can keep the emotional distress caused by your disease from interfering with the things you want to do?

**IT04S**. ¿Qué tan seguro se siente Ud. de poder evitar que algunos otros síntomas o problemas de salud que tenga interfieran con las cosas que quiere hacer?

**IT04E.** How confident do you feel that you can keep any other symptoms or health problems you have from interfering with the things you want to do?

**Total.** Items summation
